# Acute paranoid psychosis as sole clinical presentation of hepatic artery thrombosis after living donor liver transplantation

**DOI:** 10.1186/1471-2482-10-7

**Published:** 2010-02-22

**Authors:** Armin D Goralczyk, Volker Meier, Giuliano Ramadori, Aiman Obed, Thomas Lorf

**Affiliations:** 1 Department of General and Visceral Surgery, University Medical Center Göttingen, Göttingen, Germany; 2 Department of Gastroenterology and Endocrinology, University Medical Center Göttingen, Göttingen, Germany

## Abstract

**Background:**

Hepatic artery thrombosis is a devastating complication after orthotopic liver transplantation often requiring revascularization or re-transplantation. It is associated with considerably increased morbidity and mortality. Acute cognitive dysfunction such as delirium or acute psychosis may occur after major surgery and may be associated with the advent of surgical complications.

**Case presentation:**

Here we describe a case of hepatic artery thrombosis after living-donor liver transplantation which was not preceded by signs of liver failure but rather by an episode of acute psychosis. After re-transplantation the patient recovered without sequelae.

**Conclusion:**

This case highlights the need to remain cautious when psychiatric disorders occur in patients after liver transplantation. The diagnostic procedures should not be restricted to medical or neurological causes of psychosis alone but should also focus vascular complications related to orthotopic liver transplantation.

## Background

Hepatic artery thrombosis (HAT) is the most frequent arterial complication in orthotopic liver transplantation (OLT) occurring in 2.5-6.8% of adult transplant recipients including adult living donor liver transplant recipients [1-3] with an increased incidence in pediatric and ABO-incompatible liver transplantation [4,5]. The clinical presentation of HAT varies depending on the time of onset after transplantation. Severe acidosis, fever, systematic inflammatory response syndrome, or hepatic failure may ensue in early HAT (less than one month after OLT), whereas presentation of late HAT may vary from biliary complications such as recurrent biliary sepsis and stenotic lesions of the biliary tract, to an asymptomatic clinical picture with a mild elevation of serum transaminases and bilirubin levels [2,6]. HAT may thus be indicated by elevated serum transaminases, prolonged prothrombin time, or elevated total bilirubin and may be confirmed by doppler ultrasonography or angiography. Although in approximately 47% HAT requires re-OLT, cases without progressive allograft failure arterial flow may be resolved by fibrinolysis, interventional treatment, or surgical thrombectomy [1]. Mortality of HAT may vary from 55.6% (early HAT) to 15% (late HAT) [2]. The most common cause for HAT is surgical technique but hemodynamic or immunologic factors, such as reperfusion injury, hypercoagulation, and viral infections have also been recognized [1,7]. Risk factors for HAT include the number of allografts, cytomegalovirus (CMV) infection status, high recipient/donor weight ratio, biopsy proven rejection, and combination of female donor and male recipient [2].

## Case presentation

A 42-year-old male patient underwent a living-donor liver transplantation (LDLT) for progressive hepatitis B, cirrhosis with 7 points on the Child-Turcotte-Pugh scale and a model for end-stage liver disease score of 10. He was placed on the waiting list for transplantation because of suspected hepatocellular carcinoma. He had no history of psychiatric disorder. The donor was his 43-year-old brother who had no major medical problems. The clinical match was appropriate (see Table 1), there were no contraindications and after further evaluation recipient and donor were found to be suitable for LDLT.

**Table 1:** Characteristics of donor and recipient.

	Donor	Recipient	
Age	43 yr.	42 yr.	
Body weight	60 kg	73 kg	1.22*
BMI†	21	24	1.17*
Blood group	A Rh pos.	A Rh pos.	
CMV IgG‡	pos.	pos.	
HBs-Ag	neg.	pos.	
Anti-HBc	pos.	pos.	
Anti-HBs	*> *1000 mlU/ml	*<*10 mIU/ml	
Anti-HCV	neg.	neg.	

* Recipient/Donor ratio

**† **Body mass index

**‡ **Cytomegalo virus IgG status (IgM both neg.)

The donor underwent standard right lobectomy with preservation of the middle vein. In the recipient a standard total hepatectomy was performed, but due to successive intimal dissection of the hepatic and celiac artery complex arterial reconstruction was necessary. Due to the small diameter of the donor hepatic artery a direct anastomosis to the recipient supraceliac aorta was not feasible so an allogenic iliac artery conduit was required. The interposition graft was obtained from a heart-beating donor of a previous OLT and stored in histidine-tryptophane-ketoglutarate solution at +4°C for 10 days. Anastomosis of the bile duct was performed by duct-to-duct anastomosis to the donor right hepatic duct remnant.

At the time of transplantation the patient received induction immunosuppression consisting of 500 mg methylprednisolone and 20 mg basiliximab, the latter was repeated on the 4th post-operative day (POD). Immunosuppression was maintained with oral mycophenolat mofetil from the first POD at 2 × 1 g daily and oral prednisolone with an initial daily dose of 75 mg. The latter was tapered by 5 mg every two days. On the 5th POD oral tacrolimus was added to the immunosuppressive regimen starting with 0.5 mg on the first day and increasing daily dose by 1 to 2 mg to achieve trough plasma level of 4-10 *μ*g per liter. After surgery the recipient received continuous intravenous infusion of 5000 units of unfractioned heparin per day for anticoagulation. For prevention of (re-)infection the patient also received 10^3 ^units of hepatitis B immunglobuline at reperfusion and intravenous infusion of piperacillin/tazobactam at surgery until the 3rd POD but no antimycotics or prophylactic CMV therapy (both donor and recipient had experienced CMV infection).

In the postoperative course serum transaminases peaked on the first and 4th POD but then decreased continuously as prothrombin time increased (coagulation factors were not substituted). Albeit a gradual increase in total bilirubin plasma levels only a mild graft dysfunction was suspected because bile flow over the T-drain increased. Daily doppler sonography of hepatic vessels showed unaltered graft perfusion. Overall the patient had an uncomplicated postoperative without signs for infection or organ dysfunction. The clinical course and laboratory findings did show mild a graft dysfunction but neither rejection nor acute graft failure were indicated.

On the 9th POD the patient suffered from acute paranoid psychosis, being alert but agitated and physically aggressive. He was mistrustful and afraid of being harmed by people around him. The patient became unmanageable and had to be sedated with an intramuscular injection of 5 mg haloperidol and an intravenous injection of 20 mg diazepam. Due to this unusual presentation of postoperative delirium a diagnostic workup was initiated. Patent hepatic artery could not be shown in doppler sonography and subsequent angiography. Explorative laparotomy on the same day revealed a complete thrombosis of the donor hepatic artery and gross ischemic infarction in all liver segments. Because progression of infarction and subsequent graft loss was to be expected the patient was placed on the transplant waiting list for re-OLT.

**Figure 1:**
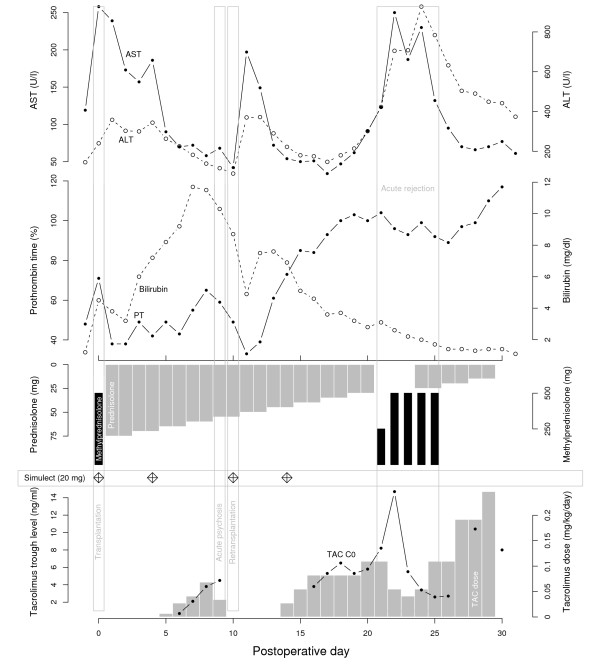
**Postoperative course of the patient**. Postoperative course of the patient. Upper panel depicts aspartate transaminase (AST, solid line) and alanine transaminase (ALT, dashed line) plasma levels in units per liter. In the middle panel total bilirubin (dashed line) and prothrombin time (PT, solid line) in activity percentage is shown. The lower panel displays immunosuppression: methylprednisolone, black bars; prednisolone, grey upper bars; basiliximab (Simulect), diamonds; tacrolimus trough levels, solid line and dots; tacrolimus dose, lower grey bars.

An acceptable organ was offered from an 18-year-old heart-beating donor on the following day and the patient underwent re-OLT 10 days after the LDLT. The hepatic artery of the new graft was reanastomosed to the old conduit. The immunosuppressive regimen was reinitiated as described above including tacrolimus (see Figure 1). Emergence from anaesthesia was uneventful and the psychiatric symptoms did not recur in the postoperative course. Furthermore a clinically suspected episode of acute rejection indicated by rising transaminases was treated with a course of methylprednisolone (see Figure 1). Daily doppler sonography of hepatic vessels showed an unaltered graft perfusion. Examination of the explant revealed thrombotic occlusion of the hepatic artery with anaemic infarction of all liver segments.

## Discussion

Here we describe a case of HAT after LDLT preceded not by signs of an liver failure but by acute paranoid psychosis. At transplantation the patient required a complex arterial reconstruction with an interposition graft which is a known risk factor for HAT [2,8]. Whether supraceliac or infrarenal placement of the interposition graft is beneficial remains equivocal [1,9]. LDLT itself has also been implied to be a risk factor for HAT, but Hatano et al could show increased incidence of HAT only in ABO-incompatible grafts [5]. In the described patient we observed a mild graft dysfunction indicated by increasing bilirubin in the first week after LDLT but accompanied by decreasing transaminases and an increasing synthetic liver function. Daily doppler sonography showed patent vessels until he suffered from acute psychosis on the 9th postoperative day.

Psychiatric disorders are not uncommon in the postoperative course of the liver transplant patient [10]. Like other patients undergoing major surgery, transplant patients may suffer from postoperative delirium. The cause of delirium is multifactorial, including predisposing factors (age, primary disease), medical/surgical factors (length of surgery, use of a cardiopulmonary bypass, water-electrolyte imbalances, sepsis, etc), and postoperative emotional stress, like prolonged sleep deprivation, use of physical restraints, and pain [11,12]. In the described patient postoperative delirium was unlikely because after surgery delirium typically evolves immediately after emergence from general anaesthesia or, after a lucid interval, in the first 72 hours [13].

Psychiatric disorders may also be drug induced. Immunosuppressive drugs like tacrolimus and corticosteroids are known causative agents of psychiatric reactions [14-16]. Neurotoxic effects are dose dependent and occur in the early phase after initiation, e.g. within the first week [14,16]. In our patient induction of the described psychiatric symptoms by immunosuppressants was also unlikely because the psychosis occurred after the first week and doses were low, i. e., 5 ng/ml tacrolimus on the 9th postoperative day (see also Figure 1). After re-transplantation he received tacrolimus and prednisolone again without any sign of recurrence of the psychiatric disorder.

Because of elevated bilirubin we also suspected impaired graft function could cause acute psychosis in our patient. In chronic liver failure manifestations of encephalopathy are common and may reach from mild obtundation with dementia and movement disorders to deep coma [17]. In the early phase of acute liver failure agitation, delusional ideas, and hyperkinesis are also common until coma finally ensues [18]. Although typical laboratory findings of acute liver failure are common, only moderate alteration, as seen in our patient, is not an exclusion criterion [19]. In our patient ammonia was also not elevated but in patients with more advanced hepatocellular dysfunction the level of ammonia is only poorly correlated with the severity of hepatic encephalopathy [17]. Besides the accumulation of unmetabolized ammonia other mechanisms have been implied as causes of encephalopathy such as imbalance of neurotransmitters, endogenous benzodiazepine ligands, or elevation of circulating tumor necrosis factor (TNF) [19,20], some of them also involved in the pathogenesis of sepsis-associated delirium [12].

Besides infection and sepsis surgical complications are the most likely cause of graft dysfunction in the early post-transplant period. Therefore we initiated further diagnostic work-up. Abdominal sonography showed no flow in the hepatic artery and subsequently HAT was confirmed by angiography. During explorative laparotomy the liver was found to be with gross infarction and we decided to evaluate the patient for re-transplantation. Luckily we obtained a graft within 24 hours and after re-transplantation psychosis resolved without sequelae.

## Conclusion

We propose that in the described patient HAT is the most likely cause of an acute liver failure although laboratory findings indicated only mild graft dysfunction. Acute liver failure was accompanied by hepatic encephalopathy apparent as acute delusional psychosis.

This case highlights the need to remain suspicious when psychiatric disorders occur in patients after liver transplantation. The diagnostic procedures should not be restricted to medical or neurological causes of psychosis but should also focus vascular complications of OLT.

## Competing interests

The authors declare that they have no competing interests.

## Authors' contributions

ADG and TL collected and analyzed the data and wrote the article. GR, HB and AO contributed substantially to the process of analyzing the data and writing the paper. All authors read and approved the final manuscript

## Pre-publication history

The pre-publication history for this paper can be accessed here:

http://www.biomedcentral.com/1471-2482/10/7/prepub
